# Expression of Concern: Long noncoding RNA HOXA-AS2 promotes non-small cell lung cancer progression by regulating miR-520a-3p

**DOI:** 10.1042/BSR-2019-0283_EOC

**Published:** 2022-07-15

**Keywords:** HOXA-AS2, Long non-coding RNA, miR-520a-3p, non-small cell lung cancer, proliferation

The Editorial Office has been made aware of potential issues surrounding the scientific validity of this paper, hence has issued an Expression of Concern to notify readers whilst the Editorial Office investigates. Duplications have been identified across multiple papers with no common authors. Figure panel 3b appears to be the same as Figure 3B of the article ‘Long noncoding RNA 00460 (LINC00460) promotes glioma progression by negatively regulating miR-320a’ (DOI: 10.1002/jcb.28232) and as Figure 3D (vertically flipped) of the article ‘STAT3-mediated upregulation of lncRNA HOXD-AS1 as a ceRNA facilitates liver cancer metastasis by regulating SOX4’ (DOI: 10.1186/s12943-017-0680-1). The authors have been contacted with regards to these concerns.

